# Prevalence of oropharyngeal dysphagia in geriatric patients and real-life associations with diseases and drugs

**DOI:** 10.1038/s41598-021-99858-w

**Published:** 2021-11-09

**Authors:** Ursula Wolf, Sandra Eckert, Grit Walter, Andreas Wienke, Sylva Bartel, Stefan K. Plontke, Christina Naumann

**Affiliations:** 1 Pharmacotherapy Management, University Hospital Halle (Saale), Ernst-Grube-Straße 40, 06120 Halle (Saale), Germany; 2 Department of Neonatology and Pediatric Intensive Care Medicine, University Hospital Halle (Saale), Halle (Saale), Germany; 3 Pediatrics, Städtisches Klinikum Dessau, Dessau-Roßlau, Germany; 4 Speech Therapist and Psychological Consultant, Practice for Speech and Language Therapy, Halle (Saale), Germany; 5 Department of Geriatrics and Geriatric Day Clinic, Diakonie Hospital, Diakoniewerk Halle (Saale), Halle (Saale), Germany; 6 Institute of Medical Epidemiology, Biostatistics and Informatics, Faculty of Medicine, Martin-Luther-University Halle-Wittenberg, Halle (Saale), Germany; 7 Department of Otorhinolaryngology, Head and Neck Surgery, University Hospital Halle (Saale), Halle (Saale), Germany

**Keywords:** Diseases, Gastroenterology, Health care, Health occupations, Medical research, Neurology, Pathogenesis, Risk factors, Signs and symptoms

## Abstract

Risk factors for oropharyngeal dysphagia (OD) in elderly patients are mainly central nervous system (CNS) and structural organic diseases or presbyphagia. We analysed the OD prevalence and association of OD with multimorbidity and polypharmacy using real-life data to complete this spectrum, with a focus on further and iatrogenic risk. This was a cross-sectional retrospective study based on a random sample of 200 patients admitted to a geriatric hospital. Data analysis included diagnoses, the detailed list of drugs, and an intense clinical investigation of swallowing according to Stanschus to screen for OD in each patient. The mean patient age was 84 ± 6.5 years. The prevalence of OD was 29.0%, without an effect of age, but a higher rate was found in men and in nursing home residents and an elevated risk of pneumonia. OD risk was slight in diabetes mellitus and COPD, and pronounced in CNS diseases. A relevant OD association was found, even after adjusting for CNS diseases, with antipsychotics, benzodiazepines, anti-Parkinson drugs, antidepressants, and antiepileptics. Further risk of OD was found with beta-blockers, alpha-blockers, opioids, antiemetics, antivertiginosa or antihistamines, metoclopramide, domperidone, anticholinergics, loop diuretics, urologics, and ophthalmics. From real-life data in patients with and without CNS diseases, we identified drug groups associated with a risk of aggravating/inducing OD. Restrictive indications for these drugs may be a preventative contribution, requiring implementation in dysphagia guidelines and an integrative dysphagia risk scale that considers all associated and cumulative medication risks in addition to diseases.

## Introduction

Swallowing is a complex, semi-automatic process that transports materials of different composition while simultaneously protecting the airways^1^. It involves 56 pairs of muscles and is performed semi-reflexively approximately 2000 times a day. Swallowing is controlled by the central nervous system (CNS) and five cranial nerves^2^. In addition, the process of oropharyngeal swallowing is even fascinatingly adapted to lifespan conditions at the anatomic level, as in newborns the epiglottis lies above the base of the tongue and food reaches the esophagus directly via the recessus piriformis on both sides of the larynx, enabling the infant to breathe and drink at the same time without choking. In contrast, the elderly need protection and prevention by identifying and eliminating any iatrogenic risk factors inducing or aggravating dysphagia.

Because of the complexity of the act of swallowing, there are heterogeneous disorders that can cause dysphagia. The main insights and most established risk factors for deterioration result from investigations of the association with underlying CNS or organic diseases, respectively. There is also increasing reference to presbyphagia^3,4^. Our serious concern is whether this defined disease entity in elderly patients neglects to adequately consider potential drug-associated risk factors.

Dysphagia is divided into oropharyngeal and esophageal dysphagia. Oropharyngeal dysphagia (OD) is a condition recognized by the World Health Organization (WHO) and defined as “the difficulty or inability to move a bolus safely and effectively from the oral cavity to the esophagus, and can include aspirations, choking, and residue”^5^. The present study exclusively relates to OD.

The objectives of our investigation were to assess the prevalence of OD in geriatric patients and analyse possible drug-related associations. Even dysphagia guidelines do not sufficiently consider this aspect in the multimorbid and vulnerable patient group, despite OD being a major complaint among elderly people^1^. OD is associated with a risk of serious complications, such as pneumonia and weight loss in advanced stages, and impairs quality of life^6^. According to an epidemiological study, approximately 5 million Germans, 7% of the overall German population, are affected by dysphagia (53% are patients in care facilities, 14% in acute hospitals, and 33% in own household)^7^. The likelihood of dysphagia in patients > 75 years of age is even higher than in acute stroke patients (50%)^8,9^. About half of nursing home residents in Germany suffer from dementia^10^.

Dysphagia is a multidisciplinary issue and has drawn the attention of physiologists, neurologists, otorhinolaryngologists, and geriatricians. This study approaches this issue from the more underrepresented pharmacological point of view, and concerning an increasing reference to the risk of an almost simplified acceptance of so-called presbyphagia. Age-related changes may affect the structure and swallowing physiology regarding mobility, coordination, and sensitivity of the swallowing process^11,12^.

Particularly these last three categories of the swallowing process may also be very susceptible to drug effects and drug side effects as indicated by increased personal awareness from > 31,200 medication reviews that included studying the content of professional drug information in detail in elderly predominantly on a polypharmaceutical regimen. Thus, to search for all risk factors associated with the manifestation of dysphagia in the most vulnerable elderly patients is necessary to obtain more complete insight and understanding of this issue.

Published data on the prevalence of dysphagia resulting from the individual patient’s medication mainly refer to single case reports. As OD is a severe, WHO-recognized burden in the elderly, all kinds of iatrogenicity should be eliminated. Polypharmacy and demographic ageing highlight the need to recognize the risk of drug-induced OD as it is an important contributor to pressure on a healthcare system under ever-increasing demand internationally. A systematic review by Attrill et al. in 2018 demonstrated the enormous impact of OD on healthcare costs in Europe and North America; overall expenditure increased by 40.36% in patients with OD, and the presence of OD added 2–8 days to hospital length of stay. Data from a meta-analysis of all cause-admission revealed an extension of 2.99 days^13^.

According to precise descriptions of side effects in the professional drug information, potential medication-induced manifestation or aggravation of dysphagia should be analysed in more detail, especially in the elderly patients’ real-life situations. Medication can influence the swallowing process by three different modalities: 1) an undesired drug effect ("normal drug side effect"), such as dry mouth (xerostomia); 2) due to a medicinal injury to the mucous membranes of the mouth, throat, and/or esophagus (e.g., from bisphosphonate therapy), requiring special drug information-appropriate conditions as a prerequisite to prevent these injuries; or 3) a complication of the desired medication effect (“complication of therapeutic action”), such as with transdermal application of scopolamine. Concerning risk insights from own 31,200 medication reviews on polypharmacy compelled this research. The studies of the specific professional drug information on each substance require a broader and carefully respective analysis of the situation under real-life conditions with regard to the concerning increased risk of potentially avoidable and even accumulative OD effects inherent with polypharmacy.

## Patients and methods

In this cross-sectional retrospective study, we analysed 200 patients admitted to the Diaconal Hospital for Acute Geriatrics in Halle (Saale), Germany. The random sample was drawn from the year 2016 following the alphabetical sorting of the patients’ medical files.

### Data collection from day of admission

Clinical swallowing was examined adapted after Stanschus^14^ (see Supplementary). We also collected information on sociodemographic characteristics (age, gender, living situation), nutritional status, Mini-Mental State Examination (MMSE), selected diagnoses/pre-existing illnesses, laboratory parameters, and individual medication lists.

### Data recorded

In each of the 200 patients, the clinical swallowing examination was performed as a comprehensive standardized non-invasive screening method by an internal speech therapist expert at day 1 of admission and documented in detail in the examination report (see Supplementary). Lack of cooperativeness and investigability as in severe cognitive dysfunction were exclusion criteria. The adapted Stanschus clinical swallowing investigation^14,15^ is based on the rating system by Daniels et al.^16^ from six clinical aspiration indicators: abnormal volitional cough, abnormal gag reflex, dysphonia, dysarthria, cough after swallow, and voice change after swallow. OD severity is assigned from 0 (normal swallowing function) to 4 (severe dysphagia). The presence of any 2 of these 6 clinical aspiration predictors have been proven to significantly distinguish patients with moderate to severe dysphagia from patients with mild dysphagia/normal swallowing^16^. The cranial nerves involved in sensory and motor testing are: facial nerve (VII) sensoric taste, motoric lip closure, jaw opening, cheek tone, salivary glands; trigeminal nerve (V) sensoric face, motoric masticatory muscles; branches of vagus nerve (X) sensoric larynx, motoric occlusion/opening of upper esophageal sphincter (not investigated here in OD), glottis closure, pocket fold activity; hypoglossal nerve (XII) motoric tongue movement; glossopharyngeus (IX) sensoric soft palate, motoric tongue, taste, salivary glands; pharyngeal plexus (IX, X) and thus investigate facio-oral motor, facio-oral sensory, and sensorimotor pharyngolaryngeal functions. Stepwise testing is performed with different consistencies: bread, followed by Jell-O and liquid in increasing amounts (see Supplementary). Cough, throat clearing, reduced cough thrust, post-swallowing, voice change are the criteria for 10 ml, 20 ml, 40 ml and 90 ml swallow test and determine categorization. All assessment data of this adapted Stanschus standardized Clinical Swallowing Examination (see Supplementary) with its individual components were recorded binäry: Patient examable/cooperative, quantitative disturbance of consciousness, qualitative disturbance of consciousness, Bell's palsy, tongue weakness, tongue movement disorder, impaired chewing function, lips reduced, inside of cheeks reduced, tongue reduced, saliva leakage from the mouth, saliva residues, food residue, abnormal voluntary cough, abnormal gag reflex, palatal elevation disturbed, dysphonia, and impaired laryngeal elevation. Cumulative identified disorders as well as severity lead to the differentiated graduation by the educated and longterm experienced speech therapist of the geriatric hospital department.

Body mass index (BMI) was assigned as underweight, normal weight, overweight, and obesity based on the classification by the WHO.

From further geriatric assessments the MMSE outcome was available in 180 patients.

Pre-existing illnesses of interest were upper gastrointestinal disorders (hiatal hernia, gastroesophageal reflux disease, gastritis, ulcers, and cancers of the lower esophagus, stomach, and small intestine), chronic obstructive pulmonary disease (COPD), diabetes mellitus type 1 and 2, CNS diseases (CNS-D; e.g., stroke, Morbus Parkinson, dementia syndrome), and documented pneumonia (acute and/or within the last 2 years if history available).

Laboratory parameters were serum albumin, serum prealbumin, serum total protein, hemoglobin, hematocrit, mean corpuscular volume (MCV), serum sodium, Quick/INR, and glomerular filtration rate (GFR). The Modification of Diet in Renal Disease (MDRD) formula that takes into account age, gender, race, and s-creatinine level was applied to define renal function according to the categorization of the National Kidney Foundation (NKF). Albuminuria could not be considered due to missing measurements. Thus, patients with normal renal function; mild, moderate, or severe renal insufficiency; or complete renal failure were differentiated. Duration of renal impairment ≥ 3 months for more accurate chronic kidney disease (CKD) classification was not investigated.

From the individuals’ medication lists, we differentiated the following drug groups for further analysis: ACE inhibitors, sartans, beta-blockers, calcium antagonists, loop diuretics, hydrochlorothiazide, other diuretics, proton pump inhibitors (PPIs), uricostats and uricosurics, urologics, chemotherapeutics, antidepressants, antipsychotics, domperidone, metoclopramide (MCP), antidementia drugs, anti-Parkinson drugs, antiepileptics, benzodiazepines, opioids, clonidine, alpha-blockers, antivertiginase and antihistamines, anticholinergics/ipratropium bromide, and ophthalmics.

### Ethics approval

The Ethics Committee of the Medical Faculty of the Martin Luther University Halle-Wittenberg approved that „No ethical concerns are raised with regard to the retrospective, anonymized data collection and analysis. Patient interests worthy of protection are not impaired by the completely anonymous collection and analysis of study data “ and that no further ethical approval is required for this retrospective study from data obtained for clinical purposes from routine care.

The Ethics Committee of the Medical Faculty of the Martin Luther University Halle-Wittenberg waived the requirement of informed consent for the entirely anonymized data collection and data analysis from all patients.

We confirm that all methods were performed in accordance with the relevant guidelines and regulations such as ethical standards of the institutional ethics committee and with the 1964 Helsinki Declaration and its later amendments or comparable ethical standards.

### Statistical analysis

The anonymized data were recorded in a Microsoft Excel file and analysed by descriptive statistics, including absolute and relative frequencies, and crosstabs using the IBM SPSS Statistic Program. Results are presented as means and standard deviations. Odds ratios (ORs), their 95% confidence intervals (CIs), and *p* values ​​were calculated by simple logistic regression to assess potential associations of individual drug groups with dysphagia. The influence of specific drug groups after adjusting for the other drug groups and the effect of a CNS disease was analysed by multiple logistic regression.

### Conference presentation

This study was presented in part at the 26th Conference of the German Drug Utilisation Research Group - German Society for Drug Application Research and Drug Epidemiology, Deutsche Gesellschaft für Arzneimittelanwendungsforschung und Arzneimittelepidemiologie, Nov 21–22, 2019, Bonn, Germany; German Congress for Patient Safety in Drug Therapy, Oct 18–19; 2018, Berlin, Germany; 14th International Congress of the European Geriatric Medicine Society, Oct 10–12, 2018, Berlin, Germany; 52th General and Family Medicine Congress - Kongress für Allgemeinmedizin und Familienmedizin, Sept 13–15, 2018, Innsbruck, Austria; 24th Nordic Congress of Gerontology, May 2–4, 2018, Oslo, Norway; 29th Annual Congress of the German Society for Geriatrics, Sept 28–30, 2017, Frankfurt/Main, Germany.

## Results

The sociodemographic data of the 200 geriatric patients revealed an almost oldest-old patient population (Table 1).

**Table 1:** Socio-demographic outline of the study population.

Patients included	200
Mean age ± SD (years)	84 ± 6.5
Females	131 (65.5%)
Living at home	158 (79.0%)
Nursing home resident	42 (21.0%)

**Figure 1:**
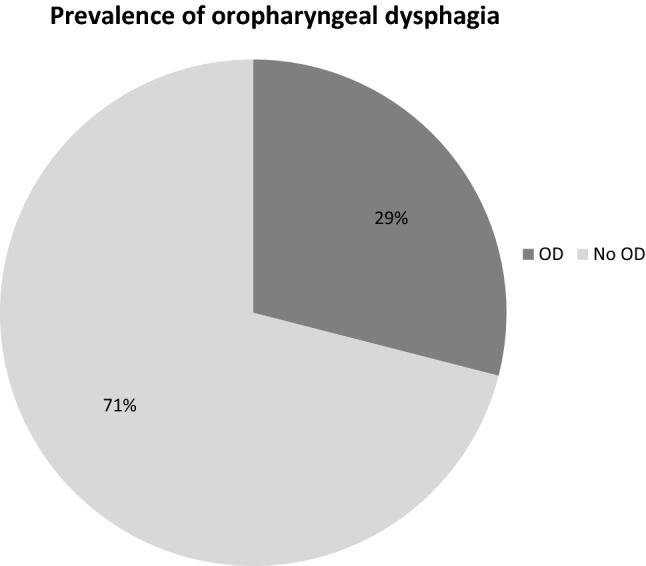
Prevalence of OD in 200 elderly patients (age 84 ± 6.5 years) admitted to an acute geriatric hospital^17^

According to the comprehensive screening investigation and individual outcomes of the applied clinical swallow examination, the prevalence of OD was 29.0% (n = 58; Fig. 1).

Nursing home residents, even though they made up only 21% of the entire study group, were more likely to suffer from OD (35.7%, n = 15) than patients who lived in their own home (27.2%, n = 43).

OD disposed a 1.5-fold elevated risk of pneumonia, OR = 1.5 (95% CI 0.65–3.58), indicating a clinically relevant association. There was no effect of age (median age of patients with OD 84 ± 6.1 years vs. non-OD patients 85 ± 6.7 years) and no association with nutritional status. Only 1 (1.7%) of the 58 OD-affected patients was underweight, whereas 10 (7.0%) of the non-OD patients were underweight. In both groups, overweight or obesity was the predominant nutritional status (70.7% in the OD group and 68.3% in the non-OD group). We did not further follow up on how long the dysphagia has been present or how the affected individuals have been treated. Low mean and median values of serum albumin and even subnormal low values of serum prealbumin and total protein were prevalent in the entire patient sample. Comparison of OD and nonOD patients revealed no additional association between OD and serum levels of albumin (37.7 ± 5.6 g/l in OD patients versus 37.6 ± 5.2 g/l in nonOD patients), prealbumin (0.17 ± 0, 06 g/l in OD patients versus 0.17 ± 0.06 g/l in non-OD patients) and total protein (64.3 ± 6.4 g/l in OD patients versus 65.2 ± 6.3 g/l in non-OD patients) measured as markers of nutritional status.

In the MMSE performed as an indicator of cognitive functional impairment, 96 (53% of 180) patients exhibited cognitive dysfunction according to MMSE categorization. Patients with severe cognitive impairment and poor cooperation and examination skills had to be excluded from OD screening because of methodology, thus we found no increased risk of OD at lower MMSE scores in the 180 measurable patients. With this exclusion category, the prevalence of the moderate to worst MMSE score was evenly distributed between OD (27.3%) and non-OD patients (27.3%), and against the same background, no increased association was found in a measured small number of patients with the lowest MMSE score and OD (2.3%, n = 1 OD patient vs 4.4%, n = 6 non-OD).

The ORs for concomitant pre-existing illnesses are given in Table 2.

**Table 2:** Association of OD with concomitant diseases.

Diseases of the upper gastrointestinal tract	OR = 0.8 (95% CI 0.43–1.67), *p* = 0.63
COPD	OR = 1.1 (95% CI 0.45–2.70), *p* = 0.83
Diabetes mellitus type 1 and 2	OR = 1.2 (95% CI 0.62–2.19), *p* = 0.65
CNS diseases	OR = 7.4 (95% CI 3.75–14.81), *p* < 0.001

In our study of OD exclusively, the esophageal dysphagia was not analyzed. There was no clinically relevant association of OD with upper gastrointestinal tract diseases such as hiatal hernia, gastroesophageal reflux disease, gastritis, ulcers, and cancers of the lower esophagus, stomach, and small intestine, which were subsumed here and not recorded separately.

As expected, the results confirm a highly associated risk with CNS diseases.

Most of the 58 patients with OD had normal kidney function (29.3%) or mild renal impairment (44.8%). Moderate renal impairment was noted in 14 (24.1%) patients and severe impairment in only 1 patient (1.7%). Compared to the entire study group (n = 195; 5 missing values), the classification was even worse, with normal kidney function in only 17.9% of patients, mild impairment in 55.9%, moderate impairment in 22.6%, severe impairment in 3.1%, and 1 patient (0.5%) with terminal renal failure.

**Figure 2:**
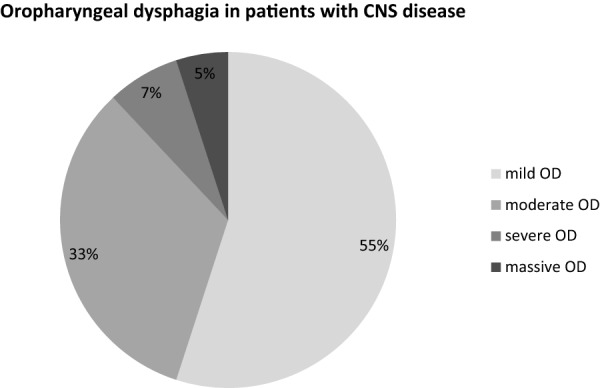
Prevalence by graduation of OD in patients with CNS disease.

**Figure 3:**
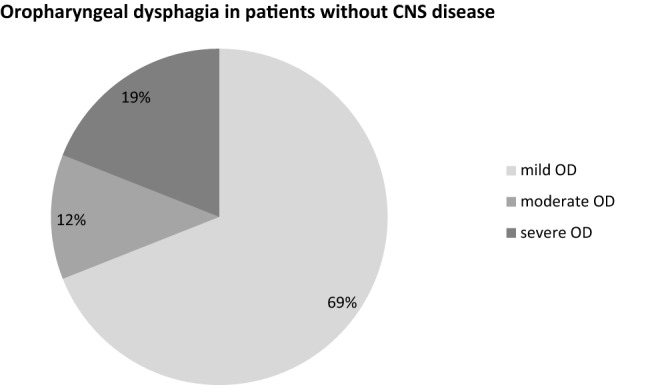
Prevalence by graduation of OD in patients without CNS disease.

Among the 58 patients affected by OD, the most prevalent category of OD was the mild form (> 50%). This was true for both the patient group suffering from CNS diseases (n = 43; Fig. 2) and patients without CNS disease (n = 16; Fig. 3). Notably, 31% of the CNS disease-free OD patients were affected by a moderate to even severe (19%) form of OD.

**Figure 4:**
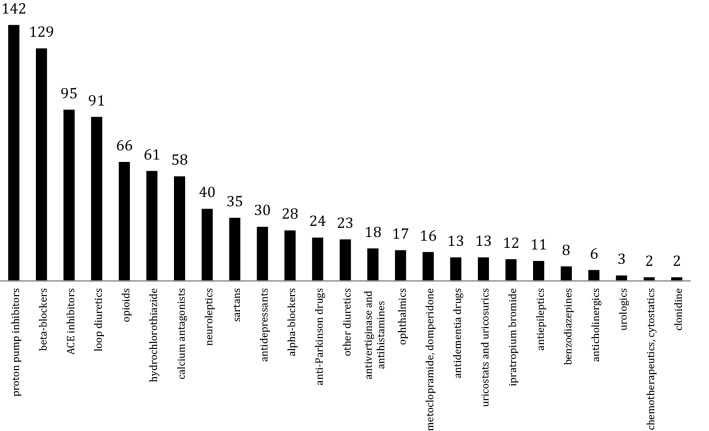
Absolute prescription frequency for the recorded drug groups among 200 geriatric patients.^17^

The detailed medication analysis revealed the absolute frequencies depicted in Fig. 4 for each drug group defined in our study.

**Figure 5:**
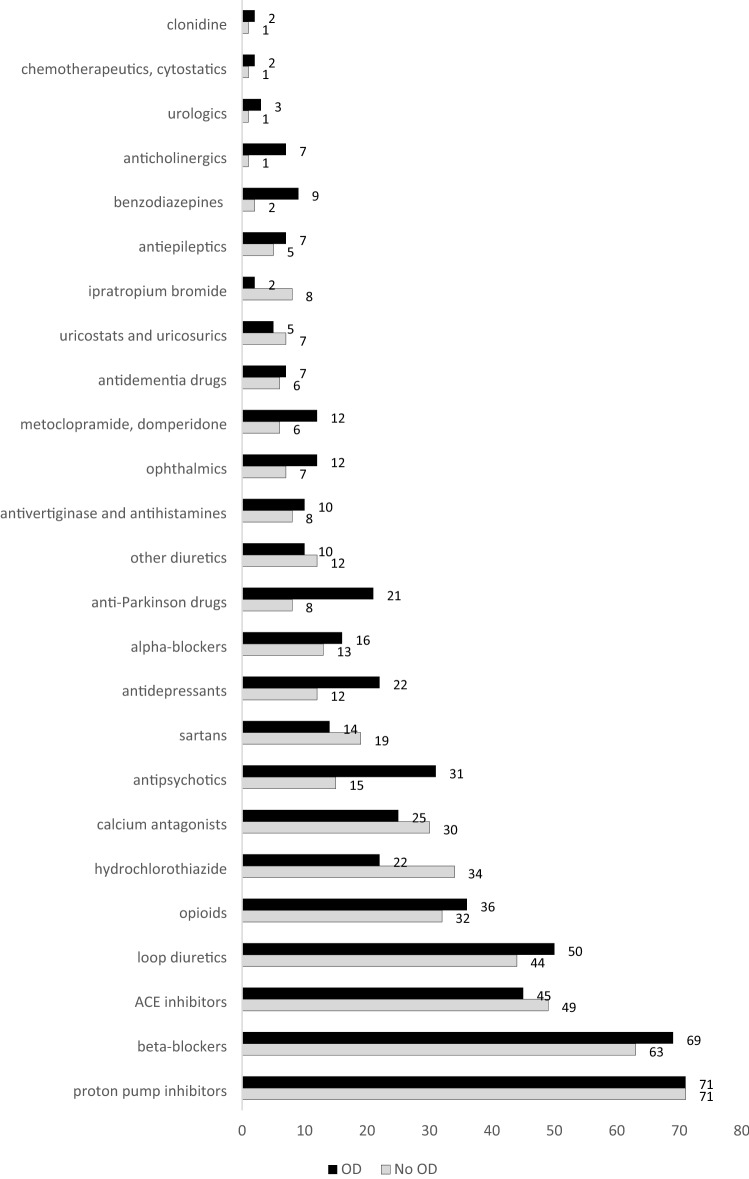
Prevalence of drug groups (%) comparing patients with and without OD.

The prevalence (%) of the investigated drug groups comparing patients with OD and without OD is shown in Fig. 5. The ORs analysed as an estimate of the effect of the defined medication groups on OD indicated a clinical relevance for beta-blockers (OR = 1.3, 95% CI 0.69–2.54, p = 0.40). The risk of associated dysphagia with loop diuretics was 1.3-times that of patients who do not consume a loop diuretic (95% CI 0.70–2.38, p = 0.42). For antidementia drugs, the effect estimate was 1.1 (95% CI 0.32–3.71), but it decreased to 0.8 (95% CI 0.21–3.01, p = 0.73) after adjusting for CNS disease. The association of OD with opioids was 1.2-fold (95% CI 0.64–2.32, p = 0.54). The association of OD with alpha-blockers was 1.2 (95% CI 0.50–2.81, p = 0.69). The risk of OD when taking urologics is 5-times the risk without urologic use (95% CI 0.45–56.65, p = 0.20), but because of the extremely low number of patients involved (n = 3), we cannot provide relevant data. The risk of associated OD was increased 1.2 when taking anticholinergics (95% CI 0.22–6.92, p = 0.81). The association of OD with clonidine was 2.5 (95% CI 0.15–40.23, p = 0.52), but only two patients were involved. Antiemetics, antivertiginosa, or antihistamines had a risk of OD 1.3-times that of patients who did not take any of these drugs (95% CI 0.45–3.51, p = 0.67). The risk OD with metoclopramide or domperidone was increased twofold (95% CI 0.72–5.73, p = 0.18). The OD association with cytostatics was 2.5-fold (95% CI 0.15–40.23, p = 0.52) but cannot be considered here due to the low number and the variety within this medication entitiy. Ophthalmics also showed an increased association (OR = 1.8, 95% CI 0.65–5.02, p = 0.25). However, they include a very heterogeneous spectrum of pharmacological agents and the low number of absolute cases did not allow a differentiated statistical analysis of the various individual active ingredients in detail; yet, an anticholinergic component is frequent.

There was not an increased association with calcium antagonists, ACE inhibitors, sartans, hydrochlorothiazide, potassium-saving diuretics, PPIs, or uricosurics or uricostatics. Hyponatremia was found in a total of 39 patients at admission (19.5%), including 7 (3.5%) OD patients. Among these 7 patients affected by OD and hyponatremia, 5 (71.4%) were on ACE inhibitors, 2 on hydrochlorothiazide, and 1 on an antidepressant, all with potential side effects of hyponatremia and xerostomia.

From the broad spectrum of medication entities studied (Fig. 5), we identified drug groups associated with a 1.4 to 4.4-fold elevated prevalence of OD (Table 3). The association was significant for antipsychotics, anti-Parkinson drugs, benzodiazepines, and antidepressants. Further adjustments for CNS diseases maintained the clinically relevant associations. The association of OD with antidepressants and antiepileptics was not even reduced after adjusting for CNS diseases (Table 3).

**Table 3:** Psychopharmacological drug groups associated with higher incidence of OD. Each group was also adjusted for CNS diseases (CNS-D).

Antipsychotics	OR = **2.5** (95% CI 1.20–5.04), *p* = 0.014
	adj. for CNS-D: OR = **1.9** (95% CI 0.88–4.31), *p* = 0.10
Anti-Parkinson drugs	OR = **2.8** (95% CI 1.19–6.73), *p* = 0.019
	adj. for CNS-D: OR = **1.3** (95% CI 0.52–3.51), *p* = 0.54
Benzodiazepines	OR = **4.4** (95% CI 1.01–18.93), *p* = 0.049
	adj. for CNS-D: OR = **2.0** (95% CI 0.42–9.50), *p* = 0.39
Antiepileptics	OR = **1.4** (95% CI 0.40–5.08), *p* = 0.58
	adj. for CNS-D: OR = **1.7** (95% CI 0.41–7.03), *p* = 0.47
Antidepressants	OR = **2.1** (95% CI 0.96–4.72), *p* = 0.06
	adj. for CNS-D: OR = **2.1** (95% CI 0.85–5.13), *p* = 0.11

## Discussion

The prevalence of OD in nearly one-third of elderly patients admitted to an acute geriatrics hospital is similar to the median value of a broad spectrum of manifestation rates referred to in the literature depending on examination methods and patient group^18–20^. The clinical screening according to Stanschus is based on expert recommendations on dysphagia management in the acute stroke phase to minimize the risk of aspiration pneumonia^21^ as a component of evidence-based guidelines with high accuracy and sensitivity^19^. The clinical swallowing examination, a German transmission from Daniels and Perry, consisting of recording aspiration and its predictors, as well as facio-oral and pharyngolaryngeal functions, demonstrated good agreement with summative clinical judgement of swallow function (kappa = 0.88)^22^.

In our study group, men were affected by OD more often than women, possibly because they tend to be transferred to hospital in more advanced stages of disease than women as documented in high-risk patients with asthma^23^. The male OD predominance could not be explained by a higher CNS disease rate.

We focused not only on pneumonia on admission, but on documented pneumonia diagnoses also within the last 2 years. From the available data, there was no differentiation for aspiration pneumonia.

Notably, there is a higher prevalence of OD in nursing home residents, who are generally more frequently on psychotropic agents than the elderly at home. Antipsychotics (47.4%), antidepressants (30.5%), opioid analgesics (28.4%), hypnotics/sedatives (22.1%), and anticonvulsants (17.9%) were found in urine samples from deceased elderly nursing home residents in a previous study by Gleich^24^. The frequent simultaneous intake of several substances with a CN effect most often is an alarming off label use^25^ and does not meet the guidelines of medical professional societies. Rösch reported that roughly half of all elderly nursing home residents have trouble with eating and often choke. Not infrequently, a percutaneous endoscopic gastrostomy (PEG) is supplied. He emphasizes that OD requires close collaboration between the otorhinolaryngologist and a speech therapist, as OD is more difficult to diagnose than esophageal dysphagia; more invasive diagnostics tools should be employed^26^. As depicted in the professional drug information, overweight up to obesity may be a result of the familiar adverse drug reactions (ADRs) of a broad spectrum of psychopharmacological agents in the elderly, such as atypical antipsychotic drugs, antidepressants, and numerous antiepileptic drugs^27^. Thus, BMI may not be affected by early and moderate forms of OD in this elderly patient group, and OD was even diagnosed in obese patients. Therefore, it is important to bear in mind the risk of drug-associated OD in these patients in competition with the masking effect of the drugs’ metabolic risks. On the other hand, geriatric patients may present cachexia and underweight not only from OD but also from gastrointestinal illnesses or esophageal dysphagia, which we did not evaluate in this study. In addition, severe or endstage cardiopulmonary diseases or cancer can explain underweight in the nonOD patients. There was no follow-up of duration or preexiting treatment in the OD patients.

Psychotropic drugs, particularly concomitant application of antipsychotics, can lead to increased appetite and obesity from their typical adverse effects, probably offsetting the effects of OD on nutritional status.

In 27% of the OD patients, there was evidence of moderate to severe cognitive impairment as indicated per MMSE scores. The main reason for the lack of association might be that patients with advanced dementia were excluded from the applied Swollow test because they did not cooperate sufficiently. Accordingly, there is no representative number of OD patients with severe dementia.

Focusing on concomitant illnesses, we found a slightly elevated risk of OD associated with diabetes mellitus and COPD, but not with the upper gastrointestinal diseases subsuming hiatal hernia, gastroesophageal reflux disease, gastritis, ulcers, and cancers of the lower esophagus, stomach, and small intestine or with severity of renal insufficiency. A study of a small sample of 20 younger patients (mean age 55 years) with chronic renal failure that used a more invasive videofluoroscopic swallowing examination revealed an almost strikingly high incidence (80%) of abnormalities in both the oral and pharyngeal phases^28^. These patients are very often on a broad spectrum of drugs that were not considered, especially antihypertensives and diuretics with anticholinergic effects causing xerostomia. Though the literature is scarce, an analysis of the co-occurrence of OD with diabetes via a simple questionnaire in younger patients found that being female and age progression were risks for OD^29^. More convincing is the diabetes-associated esophageal dysphagia, which was not integrated in our screening analysis. Esophageal motility declines with increasing duration of diabetes mellitus^30^. A 26% affection in each of two main esophageal motility parameters, the contractile front velocity and the basal lower esophageal sphincter pressure, has been demonstrated in patients with type 2 diabetes^31^.

There were no patients with cancer of the neck. Possible cervical spine diseases were not recorded and cannot be excluded. Any patients with cerebral tumors were included in the patients with CNS diseases.

In concordance with most established OD risk factors, our data confirmed the significant association with pre-existing CNS disease, predominantly such as stroke and Morbus Parkinson^32^. Yet, there is a broad gap within dysphagia prevalence, reported in a systematic review to be between 8.1 and 80% in stroke patients, almost the same with Morbus Parkinson (11–81%), but it is more concrete (27–30%) in brain injury patients after trauma. Against the backdrop of these striking leaps in prevalence, Takizawa et al. criticized the unsatisfactory situation in these most vulnerable patients, as reflected by the inhomogeneity of the diagnostic approaches, and they concluded that, despite potentially serious complications, OD often seems to be underdiagnosed and is not recognized^32^. Potentially important aspects from drug-aggravated or -induced OD suggest future consequences not only for otorhinolaryngologists, but geriatricians and general practitioners. Only a few studies from a theoretical point of view have focused on the drug-associated risk of OD in the susceptible elderly patient population^33–35^, though impressive case reports have been published^36^.

The highly elevated association of antipsychotic drugs with OD is concerning. As these neuroleptic agents induce extrapyramidal symptoms both the oral and pharyngeal swallowing phases can be affected. There have already been several case reports over the last 25 years on these drug-induced complications being reversed after withdrawal of the antipsychotics^37–40^. This does not seem to touch on the increasing prescription rate of antipsychotics in indiscriminate off-label behaviour in the elderly population. This insight is based on the author's own studies including a 1-year follow-up of off-label prescribing in nursing home residents with cognitive impairment^25^ and from the first author's ongoing daily medication reviews of geriatric patients, which add up to an extensive expertise of > 31,200 and consecutively also led to the presented study. According to the dementia guidelines and the detailed drug information, there are clear restrictions on the use of antipsychotics, as their adverse drug reactions may worsen the situation.

According to the German Drug Prescription Report in 2018^41^, 80- to 84-year-olds were prescribed 3- to 4-times as many psychotropic agents as younger patients.

Additive negative effects on adequate swallowing function may explain our real-life data results documenting the high degree of OD association with antipsychotic agents, which remained doubled even after adjusting for CNS diseases. Because of the different receptor profiles of antipsychotics, there are inherently some varying side effects. Inhibiting the dopaminergic neurotransmission of all dopaminergic pathways, the D2 blockade of antipsychotics causes extrapyramidal motor disorders, and even late irreversible dyskinesias, by interfering with the nigrostriatal system. This is especially pronounced in the highly potent first generation (typical) antipsychotics, such as haloperidol, but also in some second generation (atypical) antipsychotics, such as risperidone, and the third generations, such as aripiprazole. Early dyskinesias, such as involuntary muscular movements, and enduring throat cramps and chewing movements, called "rabbit syndrome", may affect swallowing, even restlessness while sitting, akathisia, may be a problem. On the other hand, their metabolic side effects, including weight gain, may mask less severe forms of OD^42^. The association of typical and atypical antipsychotics in elderly and community-acquired pneumonia in a dose-dependent manner soon after the beginning of treatment is explained by the risk of dysphagia from their extrapyramidal adverse events plus sedation and dry mouth with impaired oropharyngeal bolus transport as a result of action at H1 and cholinergic receptors^43,44^. Accordingly, dyskinesia of the oral pharyngeal musculature, rigidity, and spasm of the pharyngeal musculature may result in dysphagia and the CNS H1-receptor blocking, especially in the vulnerable elderly population, may cause enhanced sedation and impaired laryngeal reflexes.

Although caution is warranted in interpreting the association of OD with benzodiazepines because of the extremely small number of cases, it is apparently a clinically relevant problem that has been consistently documented in literature. In 1979, Hockman et al. described the inhibitory effect of diazepam on reflexively induced deglutition^45^. Dating back already to the 1990s, these warning delineations have remained without consequences, particularly for almost indiscriminate prescription in the most vulnerable elderly and CNS disease-affected patients. In 1997, Dantas et al. reported a case of a 57-year-old woman suffering from dysphagia after being on 2 mg lorazepam daily for 2 years. The authors documented very precisely pharyngeal retention of 47% of the volume swallowed by extended confirmation of the radiological findings via scintigraphic examination of the oral and pharyngeal phases of swallowing. Two weeks after withdrawal, the patient had no further symptoms and repeated scintigraphic analysis did not show any further retention. Chronic ingestion of benzodiazepine was concluded to potentially cause dysphagia^46^. Manometric examination of nitrazepam-induced drooling and aspiration in two children revealed that the onset of cricopharyngeal relaxation during swallowing was delayed until after the onset of hypopharyngeal contraction, indicating a drug-induced cricopharyngeal incoordination^47^. Again studies have also demonstrated an association with aspiration and pneumonia^48,49^. In a population-based propensity-matched retrospective cohort study that enrolled 7516 patients, the use of benzodiazepines was associated with a 2.21-increased risk of chronic-onset post-stroke pneumonia^48^. Although our case numbers are rather low for benzodiazepines, the results at least indicate a risk of inducing or aggravating iatrogenic OD components in CNS disease patients. It has been speculated that benzodiazepines may selectively induce pharyngeal dysphagia, probably as a result of suppression of the brainstem regulation of swallowing. Decreased arousal, impaired coordination of oropharyngeal swallowing, and oropharyngeal sensory impairment have been suggested as various mechanisms by which medications can cause OD^50^.

Our doubled estimated risk association of OD with antidepressants is concerning. Notably, this effect remained clinically relevant after adjusting for CNS diseases. Drug information refer to very frequent xerostomia and, depending on the kind of antidepressant agent, anticholinergic side effects and impaired cognition, and occasionally even extrapyramidal disorders with SSRIs such as citalopram^51^. In addition, dysarthria, taste disorders, and drowsiness may occur with tricyclics, such as amitriptyline^52^. According to the German Drug Prescription Report, there was an enormous fivefold increase, from 292 to 1467 Mio defined daily doses (DDD), in the prescription of antidepressants from 1995 to 2016^53^, reaching an exceptional position in the field of psychotropic drugs. There is no other substance group with comparable growth, although meta-analyses of antidepressant medications have reported only modest benefits over placebo treatment. Drug-placebo differences in antidepressant efficacy increase as a function of baseline severity but are relatively small, even for severely depressed patients^54,55^. Against this background, acceptance of the undesirable side effects, such as elevated OD risk, is particularly critical. In contrast, concerning the esophageal phase of swallowing, which was not focused on in our study, there are data requiring more controlled trials referring to a positive effect of antidepressants on functional esophageal disorders and chest pain^56,57^.

Antiepileptics are also among commonly prescribed centrally active agents because they are applied not only for treatment of epilepsy, but increasingly for different pain therapies, often in geriatric patients and psychiatric disorders. In our study population, even after adjusting for CNS diseases, the remaining 1.7-fold increased prevalence of OD in patients with intake of antiepileptics highlights an association. A study conducted to assess side effects of classical and new antiepileptics in 100 younger male patients suffering from intractable traumatic epilepsy revealed that dysphagia was highly common, with 20% of these patients treated with antiepileptics (mono or multiple antiepileptic therapy) over the years. Drug interactions are important to know within this drug group. Dysphagia was clearly due to the adverse effects of the antiepileptics and not by a post-traumatic CNS lesion, as withdrawal of the antiepileptic drugs resolved the impaired swallowing^58^. There are several mechanisms by which neurological side effects of antiepileptics can induce or exacerbate neurogenic OD as movement disorders, but also myopathy and disturbance of salivation. A low resting saliva flow rate found in the stroke group could be attributed to a drug-induced side effect of hyposalivation rather than an effect of stroke^59^. A review including Medline research to identify drugs associated with effects on salivary glands over a 22-year period resumed the forthcoming evidence for xerogenic drugs as most pronounced from anticholinergics with activity against the M3 muscarinic receptor, but also sympathomimetics, various antihypertensive agents, and antidepressive serotonin and noradrenaline reuptake inhibitors, and numerous further agents^60^. This is pharmacologically well established and the risks outlined in the provided professional drug information meant to be read by the prescriber. Drugs are the most common cause of reduced salivation^60^. In 1994 already, another review of the 200 most frequently prescribed drugs in the USA revealed the predominant oral ADR to be dry mouth in 80.5% of the different medications covered^61^. Twenty-three years ago, Madinier already focused on hyposalivation being particularly frequently in elderly people, with numerous drugs prescribed on a long-term basis, and in psychiatric patients. The clinical relevance is still neglected, though there is a well-known and even elevated cumulative risk in polypharmacy from antimuscarinic and antihistaminic agents, imipraminic antidepressants, and phenothiazic antipsychotics^62^ and hundreds of more drugs that may induce hyposalivation and xerostomia as referred to in their professional drug information. Bergdahl et al., in a study of 1202 subjects, measured significantly reduced unstimulated resting salivary flow among the most important variables associated with medication, such as anticholinergics and symphathomimetics, psychotropics, antiasthmatics, and diuretics^63^.

Anti-Parkinson drug application is inherently coincident with manifest Parkinson disease, which is known to cause dysphagia. Thus, as underlined by our data, OD association with these drugs is significantly reduced after adjusting for CNS disease. The entire pathophysiological mechanism of Morbus Parkinson dysphagia is ongoing examined, as both dopaminergic and non-dopaminergic alterations seem to be involved. Both aspects may also become affected by additional drug application influencing neurotransmitters with an effect on the swallowing process, as well as the patient’s alertness, with a further negative impact on OD^64^. Although OD is known to be caused by Parkinson ‘ s disease, there is evidence in the Summary of Product Characteristics (SmPC) of common adverse effects such as hyposalivation with risk of OD from standard anti-Parkinson drugs such as levodopa and carbidopa^65^, amantadine^66^, bromocriptine^67^ and rasagiline^68^, even though they may improve swallowing function at the motor level. The latter positive effects can also fluctuate and be volatile. Additionally sore throat and mouth ulcerations are common with selegeline^69^. Concerning the anti-Parkinson drugs, it will be a challenge to differentiate between anti-Parkinson drug induced effects from the disease complications themselves, whereas larger study population designs investigating the deprescribing effect concerning further psychopharmacological agents may indicate their additive effects to be avoided in Morbus Parkinson patients. The fact that approximately 80% of Parkinson patients develop dysphagia during the course of their disease^64^ should imperatively suggest avoiding further medication-related risk from other OD-associated drug groups identified.

The significant influence of masticatory function and salivation on dysfunctional swallowing has been documented in a small number of stroke patients^70^. There has not been any focus on drug effects, though all patients were on antidepressants, antiepileptics, and antihypertensives, which have the potential for hyposalivation related to their more or less pronounced anticholinergic side effects^61^. In accordance with our findings from a limited number of patients, the same is true for urological medication, which has an OR of 5.91 (95% CI 4.04–8.63) for xerostomia and salivary gland hypofunction in interventional studies in a systematic review and meta-analysis of medications that cause dry mouth as an ADR in older people^71^.

Xerostomia is also known to be a first symptom of overdose with atropine eye drops. A brinzolamide eye drop suspension can lead to dry mouth^72^, and clonidine eye drops often induce xerostomia^73^. Our data seem to confirm the association of OD with eye drops, yet the number did not allow further differentiation, though anticholinergic effects and side effects leading to dry mouth should be a main cause. Little evidence is available on this important topic. Wolff et al. stated that medications acting on almost all systems of the body may also cause side effects related to the salivary system. They compiled a comprehensive list^74^ that is supported by our own insights into potential ADRs. The spectrum of drugs involved in xerostomia is extremely broad, and it is imperative to be aware of cumulative effects, especially in the polypharmaceutical regimen of the elderly. This also concerns diuretics, ACE inhibitors, sartans, antiemetics, and antivertiginosa. For the latter, sedation from H1-receptor blocking in the CNS leads to impaired laryngeal reflexes, facilitating OD. The anticholinergic adverse effects of these and other drugs also have to consistently be taken into account in the elderly, not only in this context. It is imperative to study the provided professional drug information for knowledge of these potential ADRs and to prevent further drug-related iatrogenic swallowing dysfunction as a prescriber. The risk is exceptionally increased by cumulative ADRs within the polypharmacy of the elderly and in pre-existing swallowing disorders in CNS disease patients.

With regard to prevention and drug safety in the health care system, as well as an elderly and/or CNS disease-affected patient concern, these “OD associations from real-life data” should furthermore implicate an integrative risk score for drug-related and drug-aggravated OD. Studies on the effects of ageing on swallowing difficulties^75^ must keep in mind that these patients are mostly on polypharmacy, and this imperatively demands the medications’ intensely comprehensive and adequate inclusion within a complete dysphagia diagnosis.

### Strengths and weaknesses

This was a retrospective cross-sectional study. 200 patients was a sample of convenience. There was no personnel capacity within this data collection to increase the number further. Our preliminary results from this relatively small patient sample in terms of the number of drug groups studied and the small numbers of cases for some individual drug groups require studies on further, more extensive patient samples. However, the results are in accordance with findings from the literature. We did not collect data on the diagnosis of admission. The patients admitted to the Diaconal Hospital for Acute Geriatrics predominantly suffer from dehydration, cardiovascular decompensation, central nervous system diseases or post-operative health problems following surgical or trauma surgery interventions. Patients with sepsis as potential cause for OD^76^ are immediately transferred to the intensive care unit and thus almost certainly excluded in the study population. The geriatric-based random sample included generally admitted in-patients with a low risk of selection bias in the context of our question, as they were not specifically hospitalized for neurological reasons or dysphagia. Furthermore, the inclusion of elderly nursing home residents enhances the generalizability of the findings*.* The data collection included a broad spectrum of variables and has the limitations inherent to an observational study. Yet, there has not been any similar clinical study on this increasingly concerning and complex topic. For the most vulnerable elderly patients on polypharmacy, the study is long overdue. For broader, precise conclusions, the explorative hypotheses should be confirmed by extended study population samples to enable application of the grade framework with consideration of the Bradford Hill criteria to put forward the insights from this exposure-risk assessment in strong recommendations for public health. Focusing on the three criteria of causation and causality, we cautiously claim that a causal relationship of our data correspond exactly to (1) the drugs’ pharmacological effects, (2) the various case reports in the literature with evidence of a complete recovery of OD after discontinuation of special drugs, and (3) the net total of possible ADR described in the individual professional drug information referring to dry mouth, dyskinesias, akathisia, decreased alertness, and responsiveness up to somnolence.

The clinical OD screening in each patient at admission by an experienced speech therapist ensures a uniform basis for a homogeneous approach in analysing associations. The applied method shows robust results when compared to others from reviews on the important non-invasive diagnostic tools available, and has even caught on in high-risk stroke patients. This non-invasive clinical screening method is standardized and sensitive but might miss additional OD patients diagnosed by more invasive procedures. Increasing the study population will allow further subdifferentiation within the individual drug groups demonstrated to reveal an association with OD. Despite these limitations, the findings of drug-associated OD are concerning and support following an individualized approach. To provide more evidence and to rule out the observational study’s risk for bias as reverse causality and residual confounding could be via deprescribing trials testing effectiveness and safety of removing the identified OD associated drugs. They may even enable us to recruit patients who may benefit the most from drug deprescribing, such as elderly on polypharmacy and patients with CNS diseases, such as stroke.

There are aspects in which observational studies with a large study population may provide more relevant information than randomized controlled trials. Acknowledging further limitations, such as the risk for confounding by CNS diseases, was ruled out by adjustments. The interpretations of our findings are underlined by numerous case reports over decades and the professional drug information available to all prescribers.

### Preliminary conclusion

In the case of manifest neurological disease, but also in patients without any CNS disease, our study provides clinically relevant insights into drug-associated (aggravated or drug-induced) OD from real-life data. First, the indication for the defined drug groups, predominantly psychopharmacological agents, such as antipsychotics, antidepressants, and benzodiazepines, but also antiepileptics and further drug entities described should be examined closely and carefully in this context. In addition, the combined application of several of these OD-associated drugs, as is likely in the elderly patients on polypharmacy, may become critical in a cumulative manner. Because of the increasing risk of OD in geriatric patients, the restrictive and deprescribing approach has to be used to counteract this in a preventive and curative manner. Secondly, further differentiated analysis of the individual active ingredients within the risky substance groups should consequently try to determine a drug that is the least OD risk-associated in this regard in larger case numbers e.g. within antiepileptics. Both steps are required for geriatric patients with multimedication and OD, as well as stroke patients.

Attempting to withdraw psychotropic drugs once prescribed should be a standard procedure in OD-affected patients. The presented results accent Balzer’s^34^ presumption that, “although the disorder [dysphagia] can have several causes, the patient's medication is often overlooked as a source.” Drug-associated OD should become a compelling management issue with practical relevance, and further integrative implications required as a preventative approach in patients prior to OD manifestation and with OD.

Polypharmacy and demographic ageing highlight the need to recognize the risk of drug-induced OD, as besides the individual patient’s burden OD means an important contributor to pressure on healthcare systems under an ever-increasing demand world-wide.

### Outlook

The obligatory future aspect of prevention requires identifying any drug-related OD burden in the predominantly elder patients, who are considered vulnerable because they are most often exposed to polypharmacy. Every medical discipline treating patients with OD should take into consideration the medication risk and eminently disclose any drug-induced or drug-aggravated OD resulting not only from a single agent, but probably different drugs applied simultaneously. Most importantly, this should be done in advance of far-reaching consequences, such as PEG-tube. Primary, secondary, tertiary, and quaternary prophylaxis of OD are addressed as in patients with and without predisposing diseases, and OD needs prevention, intervention, and postvention with a maximum possible withdrawal of iatrogenic medication regarding polypharmacy.

Presumably, complications such as pneumonia, cachexia, or even indications for the most incisive and invasive measure by placing a PEG, can be prevented. This may imply profound consequences on the demographically increasing incidence of OD not only for the affected individuals, but the entire health care system because dysphagia is twice as common as diabetes mellitus.

Our findings are in line with numerous published case reports and warnings from the professional medication information referring to OD risk. We are unaware of any concerning previous research that has assessed these aspects under real-life conditions as in our patient study group. Previous investigations primarily and extensively focused on diagnostic tools for optimal measurement of dysphagia and did not distinguish separately from concomitant drug risk. There certainly remains great potential for improvement, as regarding mandatory dysphagia guidelines on drug-associated risk factors aimed at optimizing the most underrepresented preventative approach in dysphagia. In this context, the implication of an interprofessionally available dysphagia risk scale may offer more complete and better dysphagia prevention and management. Our preliminary results from real-life OD data should raise awareness of the potentially iatrogenic risk of OD induced and intensified by drugs, with the challenge of managing dysphagia by preventing iatrogenic sources via initiation of an important deprescribing trend in this context. Studies on deprescribing trials to test the effectiveness of removing OD-associated drugs would provide even more evidence and help further differentiate within the risky drug groups identified. Broad interdisciplinary and interprofessional sensitization and awareness of the featured associations may help prevent or reverse drug-induced or drug-intensified OD. The WHO highlights patient safety and essential pharmacovigilance as one of the important health agendas worldwide^77^.

## Supplementary Information

Supplementary Information.
