# Supplementary material for: Prevalence of oropharyngeal dysphagia in geriatric patients and real-life associations with diseases and drugs
Source: Sci Rep. 2021 Nov 9;11:21955. doi: 10.1038/s41598-021-99858-w (PMC8578645; doi:10.1038/s41598-021-99858-w)
Supplement: Supplementary file 1 — Supplementary Information. [file 41598_2021_99858_MOESM1_ESM.pdf]

Klinik für Geriatrie

Klinische Schluckuntersuchung

1. Anamnese			
Diagnose			
Krankheitsbeginn:			
Beginn der Schluckstörung:			
Zahnstatus:	☐ intakt	☐ schlechte Zähne	☐ Vollprothese
Bisherige Diagnostik:	☐ klin. Schlucktest	☐ Rhinolaryngoskopie	☐ Videofluoroskopie
Pneumonie:	☐ ja	☐ nein	Datum
COPD:	☐ ja	☐ nein	

2. Beeinträchtigung der Untersuchbarkeit				
Pat. untersuchbar/koop:	☐ ja	☐ tlw.	☐ nein	
Quant. Bewusstseinsstörung:	☐ wach	☐ somnolent	☐ soporös	☐ komatös
Qual. Bewusstseinsstörung:	☐ orientiert	☐ unruhig	☐ desorientiert	☐ antriebsarm
Motorische Funktion:	☐ gehfähig	☐ Rollst. mobil	☐ Bett mobil	☐ bettlägerig
Kognition/Verhalten:	☐ Neglect	☐ Aphasie	☐ Dysarthrie	☐ kogn. Beein.
Äußere ungünstige Einflüsse	☐ Lage	☐ Medi.	☐ Tubus	☐ Tracheost.

3. Klinische Testung

Klinische Testung	Hinweis auf Funktionsstörung?	nein	ja	Schluck-versuch	Hinweis auf Aspiration?	nein	Ja
Facio-orale motorische Funktionen (HN V VII XII)	a) Faziale Parese<br>b) Zungenschwäche<br>c)Zungenbewegungsstörung<br>d) gestörte Kaufunktion	☐<br>☐<br>☐<br>☐	☐<br>☐<br>☐<br>☐	**10 ml**	a) Husten<br>b) Räuspern<br>c) Reduz. Hustenstoß<br>d) Nachschlucken<br>e) Stimmveränderung	☐<br>☐<br>☐<br>☐<br>☐	☐<br>☐<br>☐<br>☐<br>☐
Facio-orale sensible Funktionen (HN V)	a) Lippen reduziert<br>b) Wangeninnenseite reduziert<br>c) Zunge reduziert	☐<br>☐<br>☐	☐<br>☐<br>☐	**20 ml**	a) Husten<br>b) Räuspern<br>c) Reduz. Hustenstoß<br>d) Nachschlucken<br>e) Stimmveränderung	☐<br>☐<br>☐<br>☐<br>☐	☐<br>☐<br>☐<br>☐<br>☐
Inspektion Mundhöhle	a) Speichelaustritt Mund<br>b) Speichelresiduen<br>c) Essensreste	☐<br>☐<br>☐	☐<br>☐<br>☐	**40 ml**	a) Husten<br>b) Räuspern<br>c) Reduz. Hustenstoß<br>d) Nachschlucken<br>e) Stimmveränderung	☐<br>☐<br>☐<br>☐<br>☐	☐<br>☐<br>☐<br>☐<br>☐
Senso-motorische pharyngo-laryngeale Funktionen	a) abnorm. willk. Husten<br>b) abnorm. Würgereflex<br>c) Gaumensegelhebung gestört<br>d) Dysphonie<br>e) beeinträchtigte Larynxelevation	☐<br>☐<br>☐<br>☐<br>☐	☐<br>☐<br>☐<br>☐<br>☐	**90 ml**	a) Husten<br>b) Erstickungsanfälle<br>c) veränderte Stimmqualität<br>d) Abbruch	☐<br>☐<br>☐<br>☐	☐<br>☐<br>☐<br>☐

4. Graduierung					
**Diagnose:**					
**Grad**	☐ 0 keine Dysphagie	☐ 1 leichte Dysphagie	☐ 2 mittelschwere Dysphagie	☐ 3 schwere Dysphagie	☐ 4 massive Dysphagie
**Penetrations/Aspirationsskala**	☐ 1	☐ 2 (Penetration)	☐ 3 ☐ 4 (Penetration)	☐ 5 ☐ 6 (Aspiration)	☐ 7 ☐ 8 (Aspiration)

5. Ernährungsplanung					
Ernährungsplang. Essen	☐ Normalkost	☐ weiche Kost	☐ Dysph.- kost ☐ fein-passiert	☐ Nahrung < 50% oral	☐ 100% Sonde
Ernährungsplang. Trinken	☐ flüssig	☐ flüssig ☐eingeschränkt	☐ angedickt	☐ Trinken < 50% oral	☐ 100% Sonde
Medikamente	☐ alle	☐ nicht flüssig	☐gemörsert mit Brei	☐ gemörsert < 50 % oral	☐ 100% Sonde
Sonde	☐ keine Sonde	☐ angepasst	☐ NGS	☐ PEG/PEJ	☐ i.v.
**6. Diagnostik- und Therapieplanung**					
Diagnostik	☐ keine	☐ erneuter klin. Schlucktest	☐ FEES (Rhino-laryngoskopie)	☐ VFS (Video-fluoroskopie)	
Therapie	☐ keine	☐ kompens.	☐ restituierend	☐ adaptierend	

Supplementary above [17], Source: Department of Geriatrics and Geriatric Day Clinic, Diakonie Hospital, Diakoniewerk Halle (Saale), Germany

Examination protocol of the applied Standardized Clinical Swallowing Assessment for OD screening adapted according to Stanschus [14, 15] including the Daniels' criteria [16] for OD grading 0-4.

Prevalence of oropharyngeal dysphagia in geriatric patients and real-life associations with diseases and drugs

Supplementary Information
